# Author Correction: The ASIC3/P2X3 cognate receptor is a pain-relevant and ligand-gated cationic channel

**DOI:** 10.1038/s41467-018-05621-7

**Published:** 2018-08-17

**Authors:** Gabriele Stephan, Lumei Huang, Yong Tang, Sandra Vilotti, Elsa Fabbretti, Ye Yu, Wolfgang Nörenberg, Heike Franke, Flóra Gölöncsér, Beáta Sperlágh, Anke Dopychai, Ralf Hausmann, Günther Schmalzing, Patrizia Rubini, Peter Illes

**Affiliations:** 1 Rudolf-Boehm-Institut für Pharmakologie und Toxikologie, University of Leipzig, Leipzig 04107, Germany; 2 Acupuncture and Tuina School, Chengdu University of Traditional Chinese Medicine, Chengdu 610075, China; 3 Neurobiology Sector, International School for Advanced Studies, Trieste 34136, Italy; 4 Department of Life Sciences, University of Trieste, Trieste 34127, Italy; 5 Institute of Neuroscience and State Key Laboratory of Neuroscience, Shanghai 100025, China; 6 Laboratory of Molecular Pharmacology, Institute of Experimental Medicine, Hungarian Academy of Sciences, Budapest, 1043 Hungary; 7 János Szentágothai School of Neurosciences, Semmelweis University School of PhD Studies, Budapest, 1043 Hungary; 8 Molecular Pharmacology, Rheinisch-Westfälische Technische Hochschule (RWTH) Aachen University, Aachen 52072, Germany

Correction to: *Nature Communications* 10.1038/s41467-018-03728-5; published online: 10 April 2018

The originally published version of this article contained an error in the name of the author Flóra Gölöncsér, which was incorrectly given as Flóra Göröncsér. This has now been corrected in both the PDF and HTML versions of the article.

