# Supplementary material for: The ASIC3/P2X3 cognate receptor is a pain-relevant and ligand-gated cationic channel
Source: Nat Commun. 2018 Apr 10;9:1354. doi: 10.1038/s41467-018-03728-5 (PMC5893604; doi:10.1038/s41467-018-03728-5)
Supplement: Supplementary file 1 — Supplementary Information [file 41467_2018_3728_MOESM1_ESM.pdf]

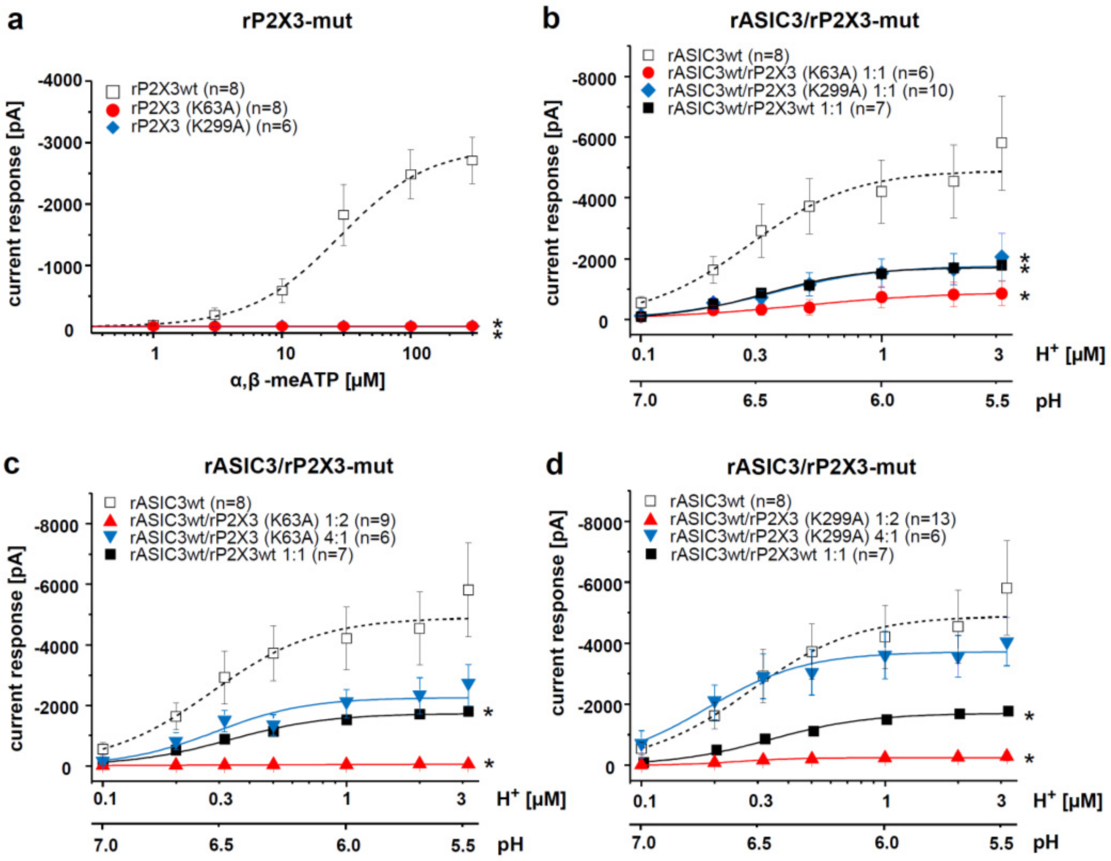

Supplementary Figure 1 Effects of binding-site mutations in rP2X3Rs on rASIC3/rP2X3R function

Whole-cell patch-clamp recordings at a holding potential of -65 mV. (**a-d**) Concentration-response curves, obtained in CHO cells, expressing the rP2X3 mutants K63A and K299A alone or together with co-transfected rASIC3wt. (**a**) Effect of α,β-meATP (0.3-300 μM) on the wt and mutated rP2X3Rs. The concentration-response curves for rP2X3 (K63A) and rP2X3 (K299A) are overlapping. (**b**) Effect of protons (pH 7.0-5.5) on rASIC3wt, rASIC3wt/rP2X3Rwt and rASIC3wt/rP2X3R-mut (1:1 transfection ratio). (**c**) Effect of protons on rASI3wt, rASIC3wt/rP2X3Rwt, and rASIC3wt/rP2X3R (K63A) (1:2, 4:1 transfection ratios). (**d**) Effect of protons on rASI3wt, rASIC3wt/rP2X3Rwt, and rASIC3wt/rP2X3R (K299A) (1:2, 4:1 transfection ratios). Means±S.E.M. of the indicated number of experiments. *P<0.05; statistically significant difference of the I_{mean} values at the highest agonist concentration from the rASIC3wt curve (one way ANOVA followed by the Holm-Sidak test).

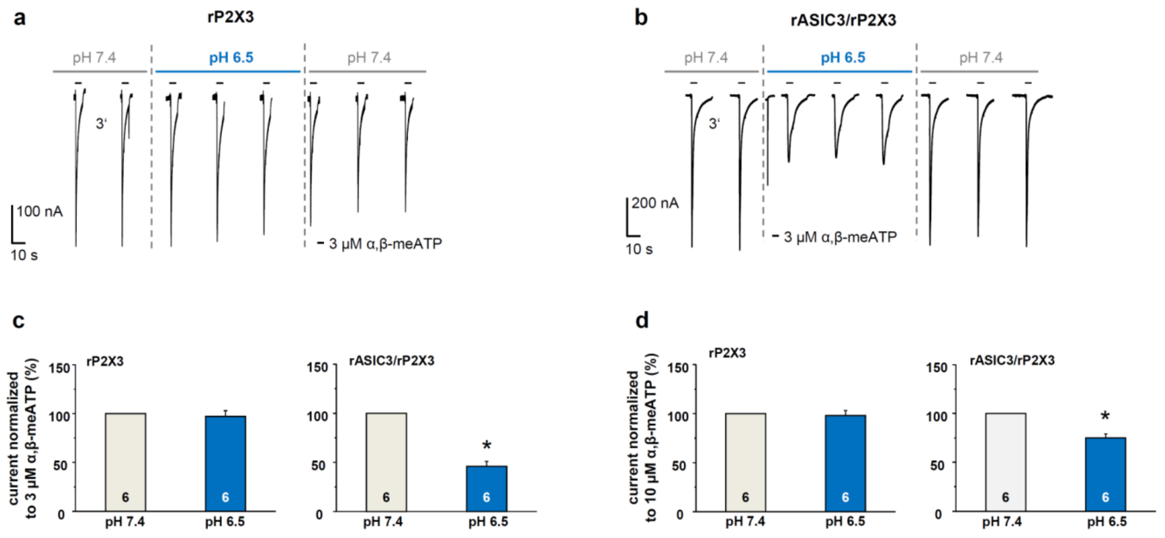

Supplementary Figure 2 Interaction between rASIC3 and rP2X3Rs after their expression in oocytes

Two electrode voltage-clamp recordings (holding potential -60 mV) of *X. laevis* oocytes expressing the His-rP2X3-StrepII (**a**) or co-expressing the His-rASIC3-EGFP and His-rP2X3-StrepII in a 1:1 ratio (**b**). Current responses were elicited by α,β-meATP (3 μM) for 5 s in 3 min intervals before, during and after a shift in pH from 7.4 to 6.5. In agreement with the functional data obtained in CHO cells (c.f. **Fig. 2 a-c**), co-expression of the His-rASIC3-EGFP produced a significant decrease of the α,β-meATP-induced His-rP2X3-StrepII current responses during acidification. Percentage changes of α,β-meATP (3 μM (**c**) or 10 μM (**d**))-induced current responses were calculated with respect to the second current before the pH shift to 6.5 (**c, d**). *$P<0.05$; statistically significant difference from the effect of α,β-meATP at pH 7.4 (Mann-Whitney test). The scale labels for the vertical bars were 100 nA (**a**) and 200 nA (**b**). The scale labels for the horizontal bars were 10 s (**a, b**).

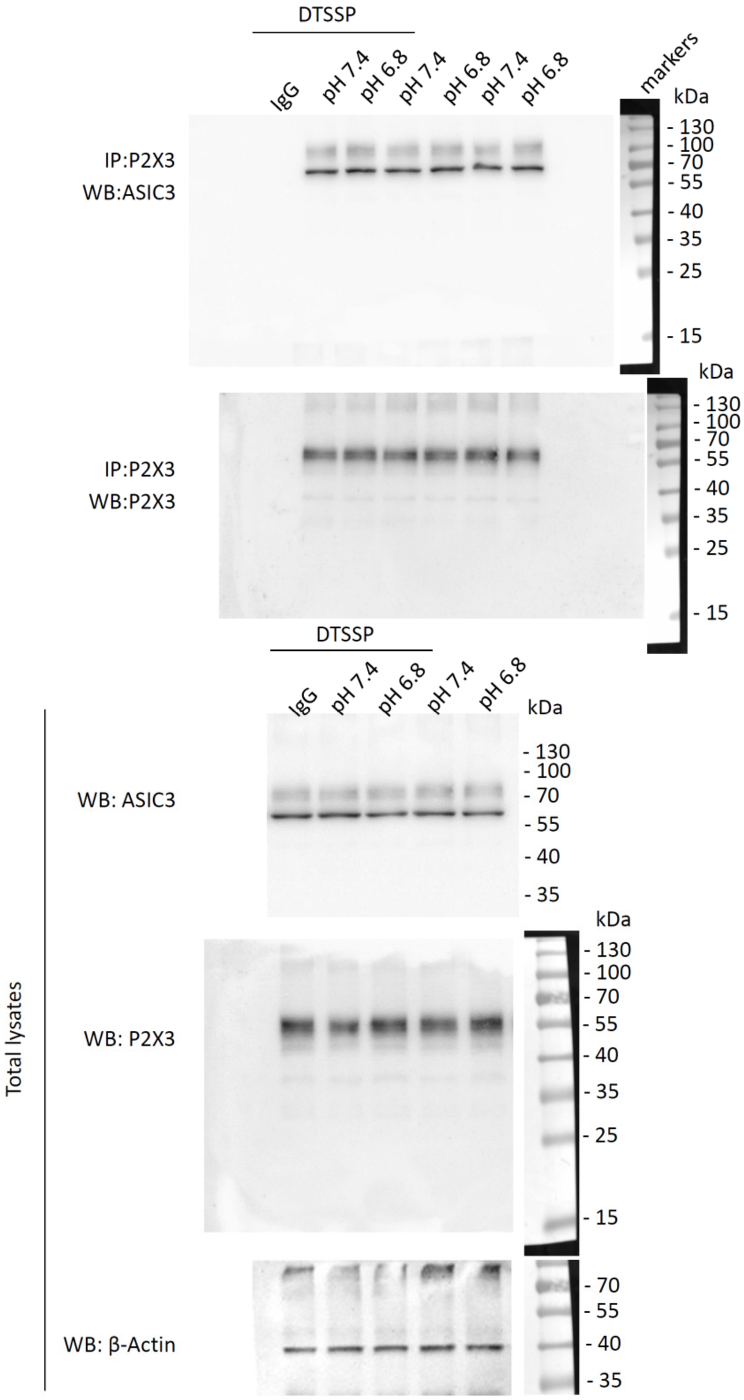

Supplementary Figure 3a Uncropped images of the cropped, displayed blots shown in Figure 8b

Raw data of immunoprecipitation (IP) of DTSSP-treated extracts of primary sensory neurons with anti-P2X3 antibodies and western blots (WB) with anti-ASIC3 antibodies. Incubation of neuronal cultures at pH 6.8 or 7.5 is indicated. ASIC3 signal is not found after immunoprecipitation with unrelated antibodies (IgG). Input ASIC3 and P2X3 contents in total extracts are also shown. β-Actin is used as gel loading control. Where available, blots with pre-stained molecular markers are also shown.

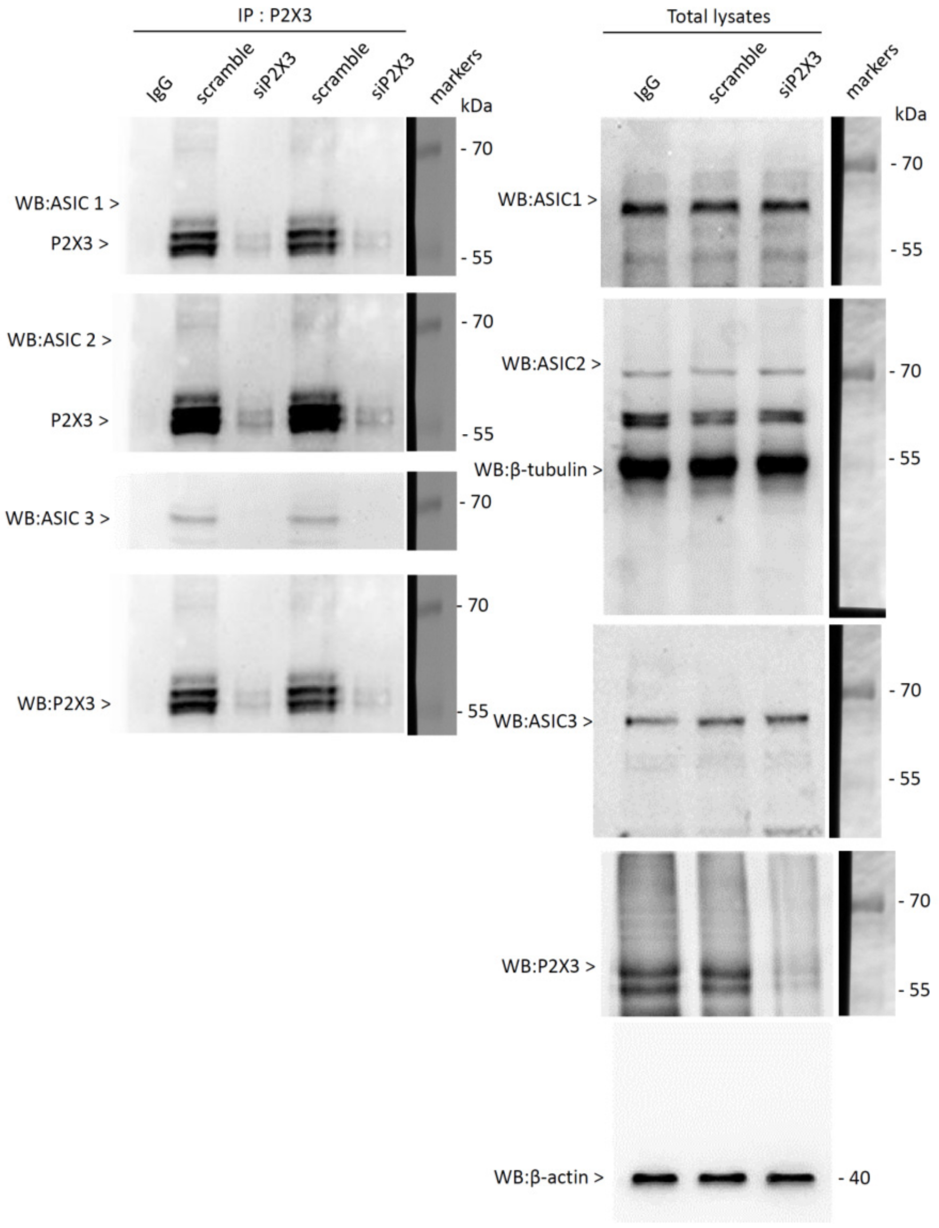

Supplementary Figure 3b Uncropped images of the cropped, displayed blots shown in Figure 8c

Raw data of immunoprecipitation of P2X3 receptors (left) with ASIC3 channels in control conditions (scramble) and after P2X3 receptor silencing (siP2X3). The same membrane from IP samples was first developed with antibodies against P2X3, ASIC1 and ASIC2 and later for ASIC3 (as indicated). No P2X3/ASIC3 signal was found after siP2X3 treatment. Western blot with anti-ASIC1 or anti-ASIC2 antibodies on the same membranes gave no signal. Pull down with unrelated antibody (IgG) gave no signal. Quality of input lysates and equal gel loading is shown (right panels, total lysates, β-tubulin and β-actin). Where available, blots with pre-stained molecular markers are also shown.

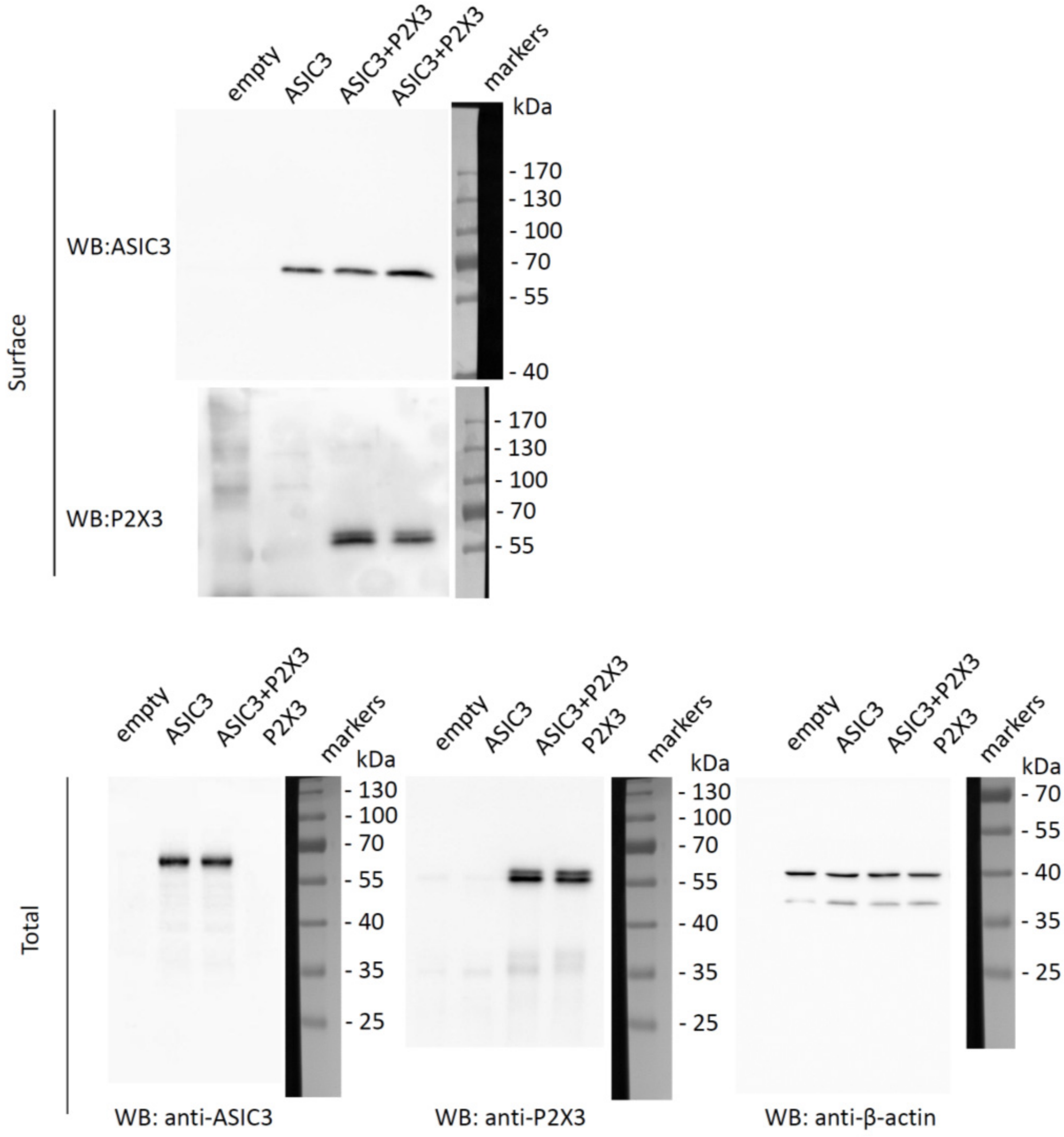

Supplementary Figure 3c Uncropped images of the cropped, displayed blots shown in Figure 8d

Raw data of membrane protein biotinylation experiments in CHO cells transfected with plasmids encoding for ASIC3 alone or ASIC3 plus P2X3 receptors (upper panels, surface). Quality of total protein extracts and controls for equal gel loading are also shown (bottom panels, total lysates). Blots with pre-stained molecular markers are also shown.

Supplementary Table 1 Data derived from Hill plots of concentration-response curves of pH changes applied onto ASIC3, or onto ASIC3 co-expressed with P2X3Rs.
Maximum current amplitudes (I_{max}), half maximal effective proton concentrations (EC_{50}; pH_{50}), Hill coefficients (n_H) and numbers of analyzed CHO cells or DRG neurons (n) are listed below. All values, except pH_{50}, represent mean ± S.E.M. [a]P<0.05; statistically significant difference from data obtained at rASIC3wt. [b]P<0.05; statistically significant difference from data obtained in the respective controls. For calculations of statistical significance, one way analysis of variance (ANOVA) was followed by the Holm-Sidak post hoc test. [&]When the curve did not reach a maximum even at 3.16 µM H^+ (pH 5.5), the experimentally determined current amplitude is indicated. n.c., not calculated.

	**I_{max} [pA]**	**EC_{50} [µM]**	**pH_{50}**	**n_H**	**n**
**CHO cells**					
**rASIC3wt**	**-6291 ± 332**	**0.25 ± 0.02**	**6.60**	**2.5 ± 0.3**	**16**
rASIC3wt + AMI	-2718 ± 327[a]	0.33 ± 0.08	6.48	1.7 ± 0.5[b]	7
rASIC3wt + A-317491	-7990 ± 748[a]	0.32 ± 0.04	6.49	2.0 ± 0.3[b]	7
rASIC3wt + [no Ca^{2+}]$_o$	-4816 ± 441	0.18 ± 0.03	6.75	1.3 ± 0.3[b]	7
rASIC3wt + [4mM Ca^{2+}]$_o$	-5879 ± 1057	0.43 ± 0.13[a]	6.36	1.8 ± 0.5[b]	6
rASIC3wt + [high Ca^{2+}]$_{in}$	-2959 ± 420[a]	0.32 ± 0.09	6.49	1.7 ± 0.6[b]	8
rASIC3wt + [low Ca^{2+}]$_{in}$	-3677 ± 420[a]	0.22 ± 0.03	6.65	2.1 ± 0.3[b]	12
**rASIC3wt/rP2X3wt 1:1**	**-1714 ± 73[a]**	**0.33 ± 0.03**	**6.48**	**2.2 ± 0.2[b]**	**7**
rASIC3wt/rP2X3wt 1:1 + A-317491	-1408 ± 169	0.39 ± 0.07	6.40	3.2 ± 0.5	8
rASIC3wt/rP2X3wt 1:1 + AMI	-703 ± 33	0.34 ± 0.02	6.47	2.8 ± 0.2	6
rASIC3wt/rP2X3wt 1:1 + 30 µM J-8	-817 ± 73[b]	0.38 ± 0.08	6.42	1.1 ± 0.2[b]	6
rASIC3wt/rP2X3 (K63A) 1:1	-913 ± 153[b]	0.40 ± 0.12	6.33	1.4 ± 0.3[b]	6
rASIC3wt/rP2X3 (K63A) 1:2	-49 ± 12[b,&]	n.c.	n.c.	n.c.	9
rASIC3wt/rP2X3 (K63A) 4:1	-2254 ± 318[b]	0.36 ± 0.05	6.54	2.3 ± 0.4	7
rASIC3 wt/rP2X3 (K299A) 1:1	-1772 ± 191	0.25 ± 0.02	6.45	2.0 ± 0.2[b]	10
rASIC3wt/rP2X3 (K299A) 1:2	-292 ± 145[b,&]	n.c.	n.c.	n.c.	13
rASIC3wt/rP2X3 (K299A) 4:1	-3723 ± 151[b]	0.23 ± 0.02	6.73	2.1 ± 0.4[b]	6
rASIC3wt/rP2X3wt 1:1 + DTT	-1092 ± 59[b]	0.30 ± 0.03	6.65	2.4 ± 0.3	6
rASIC3wt/rP2X3 (K201C/V274C) 1:1	-1480 ± 93[b]	0.30 ± 0.02	6.52	2.5 ± 0.2	7
rASIC3wt/rP2X3 (K201C/V274C) + DTT	-112 ± 2.7[b]	0.33 ± 0.03	6.53	1.5 ± 0.1[b]	7
**hASIC3wt**	**-1082 ± 165[a]**	**127.5 ± 32.5[b]**	**3.89**	**1.6 ± 0.3**	**7**
hASIC3wt/hP2X3wt 1:1	-283 ± 39[b]	45.4 ± 12.5	4.34	1.9 ± 0.8	6
hASIC3wt/hP2X3wt 1:1 + A-317491	-238 ± 46[b,&]	n.c.	n.c.	n.c.	6
**Rat DRG neurons**					
ASIC3	-827 ± 57[a]	**0.26 ± 0.03**	**6.59**	**1.9 ± 0.3**	**19**
ASIC3 + APETx2	-347 ± 90[b,&]	n.c.	n.c.	n.c.	12

Supplementary Table 2 Data derived from Hill plots of concentration-response curves of α,β-meATP applied onto P2X3 receptors, or onto P2X3 receptors co-expressed with ASIC3. Maximum current amplitudes (I_{max}), half maximal effective α,β-meATP concentrations (EC_{50}), Hill coefficients (n_H) and numbers of analyzed CHO cells or DRG neurons (n) are listed below. Values represent mean ± S.E.M. [a]P<0.05; statistically significant difference from data obtained at rP2X3wt. [b]P<0.05; statistically significant difference from data obtained at in respective controls. For calculations of statistical significance, one way analysis of variance (ANOVA) was followed by the Holm-Sidak post hoc test. [&]When the curve did not reach a maximum even at 300 µM α,β-meATP, the experimentally determined current amplitude is indicated. n.c., not calculated.

	**I_{max} [pA]**	**EC_{50} [µM]**	**n_H**	**n**
**CHO cells**				
**rP2X3wt**	**-2922 ± 221**	**27.6 ± 6.0**	**1.3 ± 0.1**	**8**
rP2X3wt + AMI	-1279 ± 343[a]	47.8 ± 39.4	0.7 ± 0.1	11
rP2X3wt + A-317491	-1202 ± 91[a]	32.3 ± 6.7[a]	1.2 ± 0.2[a]	8
rP2X3wt + [no Ca^{2+}]$_o$	-2496 ± 526[&]	n.c.	n.c.	6
rP2X3wt + [4mM Ca^{2+}]$_o$	-2225 ± 498[&]	n.c.	n.c.	10
rP2X3wt + [high Ca^{2+}]$_{in}$	-2099 ± 226	5.5 ± 2.3	1.5 ± 0.2	7
rP2X3wt + [low Ca^{2+}]$_{in}$	-2853 ± 725	50.0 ± 28.0	1.2 ± 0.1	9
rP2X3 (K63A)	-13.4 ± 3.4[a,&]	n.c.	n.c.	8
rP2X3 (K299A)	-10.1 ± 3.5[a,&]	n.c.	n.c.	6
**rASIC3wt/rP2X3wt 1:1**	**-1468 ± 129[a]**	**15.0 ± 4.2[a]**	**1.2 ± 0.3**	**6**
rASIC3wt/rP2X3wt 1:1 + A-317491	-640 ± 84[b,&]	n.c.	n.c.	8
rASIC3wt/rP2X3wt 1:1 + AMI	-621 ± 103	17.7 ± 10.9	0.7 ± 0.1	7
rASIC3wt/rP2X3wt 1:1 + J-8	-387 ± 7.2[b]	32.4 ± 1.4[b]	1.9 ± 0.1[b]	7
rASIC3wt/rP2X3wt 1:1 + DTT	-1425 ± 287	12.8 ± 6.9	1.9 ± 0.7	6
**hP2X3wt**	**-3438 ± 73**	**11.7 ± 0.8[a]**	**1.2 ± 0.1**	**6**
hASIC3wt/hP2X3wt 1:1	-3191 ± 278	23.3 ± 6.1	1.1 ± 0.2	6
hASIC3wt/hP2X3wt 1:1 + A-317491	-1051 ± 29[b]	46.7 ± 30.0	2.6 ± 0.3[b]	6
**Rat DRG neurons**				
P2X3	**-772 ± 30**	**30.9 ± 3.2**	**1.4 ± 0.1**	**11**
P2X3 + anti-NGF	-545 ± 119	48.2 ± 31.1	0.9 ± 0.3	9
P2X3 + APETx2	1381 ± 153	33.4 ± 11.9	0.9 ± 0.2	8

Supplementary Table 3 Primer sequences used for mutagenesis of rP2X3R constructs.

Primer Name	Primer sequence 5' → 3'
rP2X3R K63A for	TTGAGTCCTCAGTAGTTACCGCGGTGAAAGGCTTCGGGCGC
rP2X3R K63A rev	GCGCCCGAAGCCTTTCACCGCGGTAACTACTGAGGACTCAA
rP2X3R K299A for	CAGCGAGTACCGCACACTCCTGGCGGCTTTTGGCATC
rP2X3R K299A rev	GATGCCAAAAGCCGCCAGGAGTGTGCGGTACTCGCTG
rP2X3R K201C for	CTCACCGACAAGGATATCTGCAGGTGCCGCTTCCAC
rP2X3R K201C rev	GTGGAAGCGGCACCTGCAGATATCCTTGTCGGTGAG
rP2X3R V274C for	GTTTCTGAGAAAAGCAGTTGCTCCCCTGGCTACAACTTCAGG
rP2X3R V274C rev	CCTGAAGTTGTAGCCAGGGGAGCAACTGCTTTTCTCAGAAAC
rP2X3R I200C for	CTCACCGACAAGGATTGCAAGAGGTGCCGCTTC
rP2X3R I200C rev	GAAGCGGCACCTCTTGCAATCCTTGTCGGTGAG
[1]rASIC3-pDONR for	GGGGACAAGTTTGTACAAAAAAGCAGGCCATGAAACCTCGCTCCGGACTG
[1]rASIC3-pDONR rev	GGGGACCACTTTGTACAAGAAAGCTGGGTATCAGAGCCTTGTGACGAGGTAACAG
[2]His$_8$-rASIC3 for	GTCCGGAGCGAGGTTTGTGATGGTGATGGTGATGGTGATGAGCCATGGCCTGCTTTTTTG
[2]His$_8$-rASIC3 rev	CAAAAAAGCAGGCCATGGCTCATCACCATCACCATCACCATCACAAACCTCGCTCCGGAC
[3]His-rP2X3-StrepII for	CCTATTCTATTGGTCACAATTGGTCTCATCCACAGTTCGAAAAGTGATAGTGAATTGGGTACG
[3]His-rP2X3-StrepII rev	CGTACCCAATTCACTATCACTTTTCGAACTGTGGATGAGACCAATTGTGACCAATAGAATAGG
[4]His$_8$-rASIC3-GFP for	CACCGTACCTGTTACCTCGTCACAAGGCTCATGGTGAGCAAGGGCGAG
[4]His$_8$-rASIC3-GFP rev	CGATATCACCACTTTGTACAAGAAAGCTGGGTATCACTTGTACAGCTCGTCCATGCC

[1]PCR Gateway primer for subcloning of rASIC3 into pNKS-GW

[2,3]QuikChange primer for insertion of the indicated affinity tag sequences

[4]Megaprimer for in-frame fusion with GFP sequence
